# Habitual Constipation

**Published:** 1879-07

**Authors:** 


### EXCERPTA.

---

Habitual Constipation.—There is no derangement of the system which is more common or which remotely entails so many serious consequences as habitual constipation. The present effects, although less serious, are very unpleasant to the patient. The cerebral congestion and vertigo, which are frequetly present, give rise to a constant dread in the patient of pending apoplexy or softening of the brain. It would be impossible in the limits of a short paper even to enumerate the anomalous symptoms produced by chronic constipation. As in most diseases dependent on different causes, we should, if possible, find the exact cause, and if it still exists try to remove it. But in the greater part of such cases the original cause has long since passed away and left only its effects behind, the most important of which is the perverted habit, which takes the place of the normal habit of relieving the bowels once daily. In most cases there exists some atony of the muscular coat of the intestines and a diminished excitability. The want of success in treating these cases leads to a constant demand for new remedies, which are very fashionable for a time and are then displaced by new favorites. Of late we have the Hunyadi Janos mineral water, the Friedrichshall water. Seidlitz powders put up under new names, Cascaro Sagrado, etc. I have used some of these in suitable cases with advantage, but, as a rule, I find such success from the use of very old standard medicines, that I have no necessity to try new ones. My remedies are aloes, extract of belladonna, strychnia, comp. ext. colocynth; all used in minute doses. I rarely meet with a case of habitual constipatlon, which is not cured or entirely relieved. The aloes I use in 1-6 to 1-3 grain doses, either alone or combined with 1-30 grain strychnia, made into a silver-coated pill, and given at first morning and night. For two days no effect may be produced, but after that time the above doses will produce one or two stools per day, in the morning; consistent and without griping. If two stools are produced, I let the patient take one pill daily, at night, and after this has continued a few weeks, diminish the size of the pill gradually. The effect of small doses of aloes seems to increase with taking it; the large intestine and rectum acquire, in a few weeks, the habit of evacuating themselves daily, and frequently retain it ever after; in some cases the use of the pills must be continued months.

In a few cases I have found aloes to fail entirely and immediately, and in these cases I have succeeded in producing daily natural passages by the use of 1-15 to 1-10 grain doses of ext. belladonna, given twice a day; after a few weeks use of the belladonna it has been left off, but the bowels have continued to act regularly. I have also used one-eighth to one-quarter grain doses of comp. ext. of colocynth two or three times a day, with the effect producing daily passages, apparently natural. Many physicians may feel incredulous at the action of such minute doses, but I ask them to try them. Graham bread, fruit, both fresh and dried, many natural waters, are used continuously for years, in habitual constipation, without bad effects; they act usually as stimulants to the mucous and muscular coat of the large intestine. There is no reason why, in cases which require it, we may not give minute doses of aloes, belladonna, or extract of colocynth for many months, or even years.— *Weslern Lancet.*

